# Climate change reshapes plant trait spectrum to explain biomass dynamics in an old-growth subtropical forest

**DOI:** 10.3389/fpls.2023.1260707

**Published:** 2023-11-21

**Authors:** Anchi Wu, Xin Xiong, Roy González-M, Ronghua Li, Andi Li, Juxiu Liu, Xuli Tang, Qianmei Zhang

**Affiliations:** ^1^ Hubei Key Laboratory of Biologic Resources Protection and Utilization, Hubei Minzu University, Enshi, China; ^2^ Key Laboratory of Vegetation Restoration and Management of Degraded Ecosystem, South China Botanical Garden, Chinese Academy of Sciences, Guangzhou, China; ^3^ Lushan Botanical Garden, Chinese Academy of Sciences, Jiujiang, China; ^4^ Programa Ciencias Básicas de la Biodiversidad, Instituto de Investigación de Recursos Biol ógicos Alexander von Humboldt, Bogotá, Colombia; ^5^ Department of Biology, Faculty of Natural Sciences, Universidad del Rosario, Bogotá, Colombia; ^6^ College of Natural Resources and Environment, South China Agricultural University, Guangzhou, China

**Keywords:** carbon sequestration, compensatory growth, demographic rates, trait probability density, extreme drought, subtropical forest

## Abstract

Climate change leads to novel species interactions and continues to reshuffle ecological communities, which significantly declines carbon accumulation rates in mature forests. Still, little is known about the potential influence of multiple global change factors on long-term biomass dynamics and functional trait combinations. We used temporal demographic records spanning 26 years and extensive databases of functional traits to assess how old-growth subtropical forest biomass dynamics respond to various climatic change scenarios (extreme drought, subsequent drought, warming, elevated CO_2_ concentrations, and windstorm). We found that the initial severe drought, subsequent drought and windstorm events increased biomass loss due to tree mortality, which exceeded the biomass gain produced by survivors and recruits, ultimately resulting in more negative net biomass balances. These drought and windstorm events caused massive biomass loss due to tree mortality that tended towards acquisition species with high hydraulic efficiency, whereas biomass growth from survivors and recruits tended to consist of acquisition species with high hydraulic safety. Compensatory growth in this natural forest provided good explanation for the increase in biomass growth after drought and windstorm events. Notably, these dominant-species transitions reduced carbon storage and residence time, forming a positive carbon-climate feedback loop. Our findings suggest that climate changes could alter functional strategies and cause shifts in new dominant species, which could greatly reduce ecological functions and carbon gains of old-growth subtropical forests.

## Introduction

Climate change has potentially large consequence for ecosystem composition and structure (Allen et al., 2010; Aleixo et al., 2019; Zhao et al., 2022; Liu et al., 2023), thereby affecting local and global net carbon balances (Reichstein et al., 2013; Yang et al., 2022). However, demographic changes are critical for understanding forest carbon balance and its response to interannual climatic variations (Lewis et al., 2009; Pan et al., 2013; Chen et al., 2016; Luo et al., 2019). Net changes in aboveground forest biomass are modulated by a combination of three demographic processes, including biomass growth of surviving trees, biomass increase from recruitment of new stems, and biomass loss due to mortality (Brienen et al., 2015; Hisano et al., 2019; González-M et al., 2020). Changes in net biomass can be attributed to either a faster temporal increase in tree mortality than growth, or increased mortality accompanied by decreased growth due to temporal decreases in water availability, persistent increases in air temperature and CO_2_ concentrations, and the occurrence of climatic extremes (Breshears et al., 2005; van Mantgem et al., 2009; Ma et al., 2012; Brienen et al., 2015; Greenwood et al., 2017; O’Brien et al., 2017; Anderegg et al., 2020). Species-rich subtropical forest ecosystem, as a particularly important biome in the forest regions of East Asia, is pivotal for regulating the global carbon and water cycles (Yu et al., 2014; Wu et al., 2022). However, few studies have reported the long-term responses of net biomass changes and their three components to climatic perturbations in species-rich subtropical communities.

Systematic variations in demographic processes that underpin community assembly and dynamics over time have not provided underlying mechanistic explanations to generalize interspecific variations in response to both biotic and abiotic stresses (McGill et al., 2006; Meiners et al., 2015; Li et al., 2023). Plant functional traits that reflect life-history strategies and respond to environmental change could address this knowledge gap (McGill et al., 2006; Westoby and Wright, 2006; Adler et al., 2014; Swenson et al., 2020). Plant traits impact fitness indirectly via influencing survival, growth, and reproduction (Lavorel and Garnier, 2002; Violle et al., 2007), and can effectively predict various ecosystem processes and services (Wright et al., 2004; Wright et al., 2005; Shipley et al., 2006; Laughlin, 2014; Reich, 2014; González-M et al., 2020; Chacón-Labella et al., 2022). Environmental conditions alter ecological trade-offs and adaptive strategies associated with interspecific trait combinations, and the fitness and amount of trait variability are contingent on the nature and magnitude of environmental drivers (McGill et al., 2006; Díaz et al., 2007; Haddad et al., 2008; Mouillot et al., 2013; Adler et al., 2014; Bruelheide et al., 2018; Yang et al., 2018; Sarker et al., 2021; Iqbal et al., 2023). Herein, applying trait-based approaches to study the functional responses of most coexisting tree species to ongoing climate change and how these responses translate into changes in biomass carbon sinks could improve our ability to predict forest changes under future climate change scenarios (McDowell et al., 2008; González-M et al., 2020; Caleño-Ruiz et al., 2023).

Ecophysiological trait trade-offs that define fundamental niche differences and fitness among species in response to environmental conditions can be used to understand species coexistence mechanisms and ecosystem processes (Sterck et al., 2011; Craven et al., 2015; Aguirre-Gutierrez et al., 2019; Ge et al., 2019; Poorter et al., 2019; González-M et al., 2020; van der Plas et al., 2020). In species-rich forests, variations in multiple traits are organized along two main dimensions corresponding to trade-offs in resource acquisition-conservation and hydraulic safety-efficiency (Wright et al., 2004; González-M et al., 2020). Conservative species with high wood density exhibit high levels of hydraulically safe tissues and mechanical stability, plant survival, shade and drought tolerance, as well as better defense against herbivores, fungi, and pathogens (Van Gelder et al., 2006; Chave et al., 2009; Méndez-Alonzo et al., 2012). Consequently, they are also less vulnerable to hydraulic failure, have a lower mortality risk, may keep their stomata open and maintain a positive carbon-gaining capacity even under dry conditions (McDowell et al., 2008; Poorter, 2008; Quero et al., 2011). In contrast, acquisitive species with low wood density are related to larger xylem vessels, higher hydraulic conductivity, and higher photosynthetic rates and carbon gains (Coley, 1983; Santiago et al., 2004; Markesteijn et al., 2011; Méndez-Alonzo et al., 2012; Yin et al., 2023). Under drought conditions, it is expected that acquisitive species close their stomata and reduce transpiration to avoid hydraulic failure and cavitation of the xylem water column, thereby limiting the ability of plants to supply water to leaves for photosynthetic gas exchange (Hetherington and Woodward, 2003; McDowell et al., 2008; Quero et al., 2011). However, it is still not entirely clear how environmental factors determine the two-dimensional spectrum of plant form and function (Wright et al., 2010; Joswig et al., 2022).

Understanding the change patterns in the structure and function of forest ecosystem during and after multiple climate events such as droughts, storms, floods, fire, and lightning strikes, as well as exploring their underlying mechanisms, are crucial for forecasting forest ecosystem function and dynamics under global climate change. Therefore, there is a need for a general theory that can be applied to specific cases. Compensatory growth refers to the accelerated growth response of plants to damage, thereby offsetting adverse effects, restoring functionality, and maintaining their original growth state after perturbations (Mcnaughton, 1983; Belsky, 1986). It also appears as a common phenomenon in biology, and can provide a unique perspective to explain diverse forest growth patterns after partial mortality, including three forms: under-compensation, exact-compensation, and over-compensation (Li et al., 2021). The form of compensatory growth can be applied to explain changes in various forest growth indicators such as leaves, productivity, biomass, density, fecundity and recruitment (Jutila and Grace, 2002; Rea and Massicotte, 2010; Ascoli et al., 2019; Li et al., 2021). In nature forests, various factors could influence the pattern of forest growth over time. The responses and growth rates of certain tree species may vary significantly when site conditions change (light, soil nutrients, water and space, etc). However, detecting compensatory growth patterns and status after experiencing a period of unfavorable conditions in forests is important for the forest sector in designing future research strategies (Li et al., 2020; Li et al., 2021; Li et al., 2022).

The response trajectories and mechanistic explanations of subtropical forests to climate change remains poorly understood, partly due to the scarcity of highly replicated chronosequence data for numerous tree species during and following extreme climatic events. Over the past three decades, subtropical forest ecosystems have been threatened by various environmental fluctuations, such as severe droughts, storms, ongoing warming, and elevated CO_2_ concentrations (Pan et al., 2013; Zhou et al., 2011; Fauset et al., 2012; Zhou et al., 2014; Li et al., 2015). Of them, old forest trees with various ages, sizes and ontogenies cannot stably coexist over time, yet any large and persistent changes to the demographic processes over a short period could be the consequence of exogenous environmental changes (van Mantgem and Stephenson, 2007; van Mantgem et al., 2009). Old forests are irreplaceable, which has important implications for predicting forest vegetation dynamics. In a subtropical monsoon evergreen broad-leaved forest that is over 400 years old, we utilized repeat census data for a 1-ha permanent plot spanning 26 years and a dataset of 11 functional traits from 69 coexisting tree species (> 4,330 stems) to evaluate the long-term biomass dynamics and biomass-related plant trait spectrum space in response to multiple climatic scenarios. Specifically, we aimed to address two questions: (1) How have multiple climate stresses alter community biomass assembly and dynamics in an old-growth subtropical forest over the past two decades? (2) In the presence of multiple climatic stresses, can shifts in the plant functional trait spectrum space elucidate biomass assembly dynamics and the underlying mechanisms within this forest? We hypothesize that compared to favorable environmental conditions, higher climatic stresses have a greater impact on biomass dynamics and functional trait combinations.

## Materials and methods

### Study site and tree censuses

This study site was located in the Dinghushan Biosphere Reserve (20°09′21″–23°11′30″N, 112°32′39″–112°35′41″E) approximately 84 kilometers west of Guangzhou, in Guangdong Province, southern China. The reserve was established in 1950 to protect the natural monsoon evergreen broad-leaved forests in the southern subtropics and was accredited as China’s first national natural reserve in 1956. This region belongs to a typical southern subtropical monsoon climate with distinct dry and wet seasons. Annual average precipitation oscillates between 1,099 and 2,221 mm with a mean of 1,653 mm (based on data from 1960–2020), of which nearly 80% falls during the wet season (April–September) and the rest 20% falls during the dry season (October–March). The mean temperature is 9.6 °C in January and 30.7 °C in August with an annual mean of 22.4 °C. The site has red and yellow soils developed from the bedrock that consists of sandstone and shale. Soil clay, silt, and sand contents are 18.5%, 66.0%, and 15.6%, respectively, with a soil pH of 4.1 and an Fe content of 5.4 g kg^–1^ (Yu et al., 2019).

A 1-ha long-term permanent plot was established in the center of the reserve at an altitude of 200–300 m with a south-facing slope of 25–30° for long-term forest monitoring. The forest community is a species-rich old-growth subtropical lowland evergreen forest that has remained undisturbed for at least 400 years (Zhou et al., 2006). Since the establishment of the permanent sample plot, tree censuses have typically been carried out every ~5 years. All freestanding woody stems ≥1 cm diameter at breast height (DBH, measured at 1.3 m height) were identified, tagged, measured, and mapped using standardized census protocols. This study utilized six census data for this permanent sample plot (1994, 1999, 2004, 2010, 2015 and 2020).

### Climate change drivers

Based on 26-year records, we used the mean annual temperature (MAT), annual precipitation (AP), standardized precipitation and evapotranspiration index (SPEI), atmospheric CO_2_ concentration, and two extreme climatic events to assess the long-term changes in multiple biomass dimensions and their related plant trait spectrum space. MAT and AP data were obtained from the local weather station. The CO_2_ concentrations were derived from the Mouna Loa System Research Laboratory in Hawaii (http://www.esrl.noaa.gov/gmd/ccgg/trends/co2_data_mlo.html). The monthly-scale SPEI used in this study was extracted from the global drought monitor dataset at a 1° resolution from 1960–2020 to calculate the SPEI (Beguería et al., 2010; Vicente-Serrano et al., 2010). Both AP and SPEI values reached their lowest points in 2003 during the study period. Ciais et al. (2005) reported that an extreme global drought occurred in 2003. In addition, this permanent plot was observed to be affected by the windstorm “Super Typhoon Mangkhut” on August 16, 2018, by combining drone aerial images before, during, and after the windstorm.

### Functional traits

We selected five individual trees per species and measured 11 functional traits from 345 populations of 69 tree species belonging to 36 families. We selected sun-exposed branches and mature leaves from the designated trees to measure various traits. The 69 species accounted for more than 98% of the individuals and standing biomass in this plot from 1994 to 2020. The following multiple traits were used to describe the adaptation strategies of multiple species to environmental changes: specific leaf area (SLA, cm^2^ g^–1^), leaf nitrogen concentration (N_L_, mg g^–1^), leaf phosphorous concentration (P_L_, mg g^–1^), leaf N:P ratio (N:P_L_, mg g^–1^), photosynthetic capacity at maximum CO_2_ assimilation rates (A_sat_, μmol m^–2^ s^–1^), stomatal conductance (*g*_s_, mol m^–2^ s^–1^), sapwood-specific hydraulic conductivity (*Ks*, kg m^–1^ s^–1^ MPa^–1^), leaf-to-sapwood area ratio (A_L_/A_S_, m^2^ mm^–2^), water potential turgor loss point (TLP, MPa), predawn leaf water potential (ψ_PD_, MPa), and wood density (WD, g cm^–3^).

### Aboveground biomass and biomass changes

In any point in time, we estimated aboveground biomass (AGB, t ha^–1^) for each species of this permanent plot by using DBH-based allometric biomass equations for the stems, branches, and leaves, respectively (Wen et al., 1997). Between two successive censuses, biomass growth of survivors (BGS, t ha^–1^ yr^–1^) for each species was calculated as the annual biomass increment resulting from the growth of all surviving trees from the first census (T_0_) to the last census (T_1_). Biomass growth of recruits (BGR, t ha^–1^ yr^–1^) for each species was estimated as the annual biomass increment of trees that reached at least 1 cm DBH in T_1_ and were not sampled in T_0_. Biomass mortality (BM, t ha^–1^ yr^–1^) for each species was estimated as the biomass of all died trees between T_0_ and T_1_. Net biomass change (NBC, t ha^–1^ yr^–1^) for each species was calculated as the difference between biomass gain (BGS + BGR) and biomass loss (BM) in both censuses. Positive changes in net biomass (NBC^+^) indicated net biomass gain, while negative changes in net biomass (NBC^−^) indicated net biomass loss.

### Data analyses

Firstly, to describe changes in the functional trait space, we performed principal component analysis (PCA) with multiple traits at the individual level using the ‘princomp’ function in R. We selected the first two PC axes to create a two-dimensional trait space. The ordination of species across this surface presents a two-dimensional continuum, integrating ecological strategies in eleven trait combinations. To effectively visualize the functional spectra as density areas of species with ‘peak-valleys’, we used trait probability density (TPD) approach to calculate the occurrence probability of given combinations of trait values and employed two-dimensional kernel density estimation to construct color gradients and contour lines. For each scenario, we extracted contours at the 0.5 and 0.99 quantile thresholds of the probability distribution, thus highlighting the regions of the highest and lowest trait occurrence probability. The color gradient illustrates regions where the relative density of trait combinations is expected to increase (red) or decrease (yellow) as a result of functional transitions. These analyses were performed using the ‘TPD’ and ‘ks’ R packages (Chacón and Duong, 2018; Carmona et al., 2019).

Secondly, to quantify the amount of plant trait spectrum spaces (functional richness, FR) occupied by biomass dimension (i.e. aboveground biomass, demographic biomass and net biomass changes) in response to the TPD, we estimated the functional space using the sum of each biomass dimension’s hypervolumes and considering their probability distributions for values above 0 (Carmona et al., 2016; Carmona et al., 2019). In parallel, similarly to the use of β-diversity to determine the pairwise dissimilarity of species composition (Baselga, 2010). We used an overlap-based functional dissimilarity (β_O_) index to quantify the dissimilarities on the trait spectrum space occupancy between biomass dimensions. The β_O_ index ranges from 0, when two units are functionally identical (overlap = 1), to 1, when there was no functional overlap between them (Carmona et al., 2019). To verify the reliability of the results for the variations in trait spectrum space, we randomly selected 35 species (close to half) during each census period and performed 999 iterations to estimate the functional trait space and functional dissimilarity differences in biomass dimensions. All statistical analyses were performed in R version 3.5.3 (R Core Team, 2019).

## Results

### Long-term variability of atmospheric CO_2_, temperature, precipitation, and SPEI

**Figure 1:**
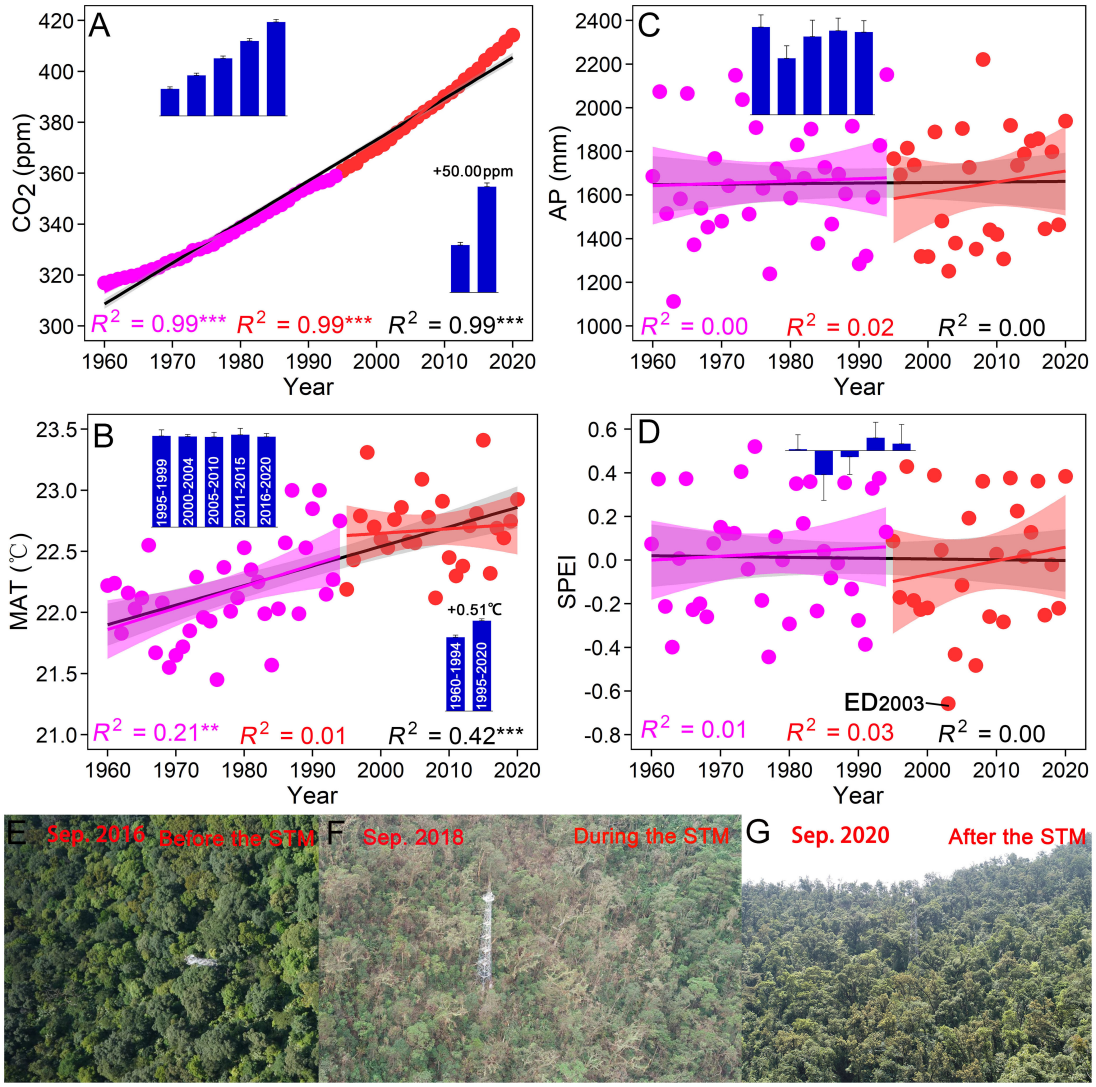
Long-term climatic changes from 1960 to 2020. **(A)** Atmospheric CO_2_ concentration. **(B)** Mean annual temperature (MAT). **(C)** Annual precipitation (AP). **(D)** Standardized precipitation and evapotranspiration index (SPEI). **(E–G)** Forest landscape before, during and after the windstorm in 2018. Bar graphs show changes in climatic indices during different census periods (mean ± SE). ED_2003_ and STM_2018_ are respectively the extreme drought in 2003 and the windstorm “Super Typhoon Mangkhut” in 2018.

Atmospheric CO_2_ increased constantly from 1960 to 2020 (*R* ^2^ = 0.99, *P* < 0.001), and increased persistently by 2.13 ppm yr^–1^ from 1995 to 2020 (ranging from 358.96 to 414.24 ppm, **Figure 1A**). MAT increased significantly from 1960 to 2020 (*R* ^2^ = 0.42, *P* < 0.001). More specifically, MAT increased significantly from 1960 to 1994 (*R* ^2^ = 0.21, *P* < 0.01), with an average rise of 0.20 °C per decade. MAT showed a non-significant upward trend from 1995 to 2020 (*R* ^2^ = 0.01, *P* = 0.66), with an approximate increase of 0.5 °C compared to the period from 1960 to 1994 (**Figure 1B**). During the period from 1960 to 2020, there were no significant trends observed for AP (*R* ^2^ = 0.00, *P* = 0.86) and SPEI (*R* ^2^ = 0.00, *P* = 0.95). The study plot experienced frequent and severe droughts from 1999 to 2011, with the AP falling below 1,500 mm in 9 out of 13 years (**Figure 1C**). The period from 2000 to 2004 was characterized as the driest, with the lowest average AP and SPEI values. Among these dry years, the lowest AP and SPEI values occurred in 2003, with values of 1,251.80 mm and –0.66 (a monthly SPEI < –0.5 indicates extreme drought), respectively (**Figures 1C, D**). Furthermore, the windstorm in 2018 also posed a threat to the forest community (**Figures 1E–G**).

### Aboveground biomass dynamics and its trait space in response to climate change

**Figure 2:**
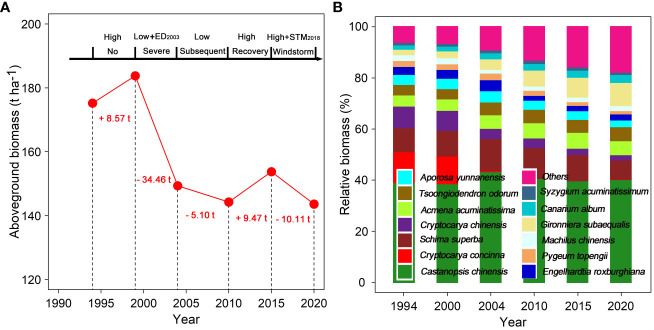
Long-term changes in aboveground biomass **(A)** and species biomass proportion **(B)** in a 1-ha forest plot from 1994 to 2020. The figure font indicates the difference in aboveground biomass between the next census and the last census. ED_2003_ and STM_2018_ indicate respectively extreme drought in 2003 and the windstorm “Super Typhoon Mangkhut” in 2018. Low and high indicate low and high rainfall in each census period.

During the period of high rainfall (1995–1999), the total AGB in the permanent study plot increased by 8.57 t ha^–1^. During the period of initial severe drought (2000–2004), especially the impact of the extreme drought in 2003, AGB decreased by 34.46 t ha^–1^. During the subsequent drought period with low rainfall (2005–2010), AGB continued to decline by 5.1 t ha^–1^. During the recovery period with high rainfall following two drought events (2011–2015), AGB increased by 9.47 t ha^–1^. However, during the windstorm disturbance period in 2018 (2016–2020), AGB decreased significantly by 8.6 t ha^–1^ (**Figure 2A**). Simultaneously, the proportion of accumulating biomass in dominant species persistently declined since the first census, while that of other subordinate and rare species significantly increased (**Figure 2B**; **Supplementary Table S1**).

**Figure 3:**
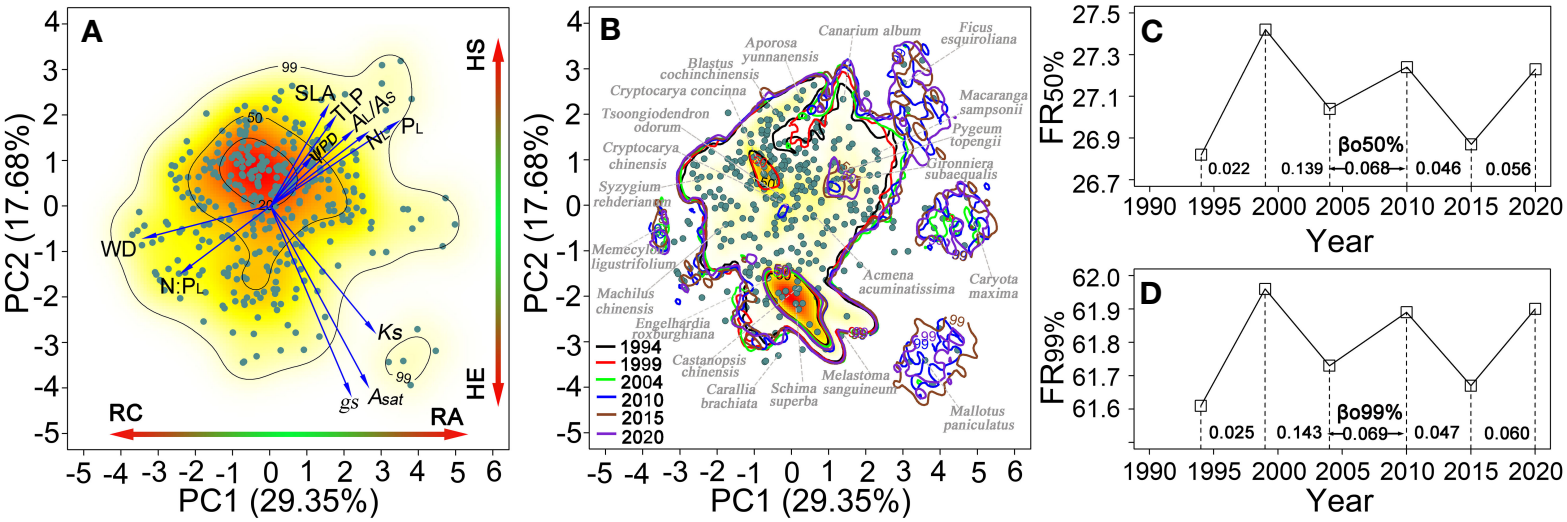
Trait probability densities (TPD) show the functional trait combinations of species populations along an axis of resource acquisition-conservation (PC1, 29.35% explained variance) and an axis of hydraulic safety-efficiency (PC2, 17.68% explained variance). **(A)** TPD where 69 tree species in this plot has an equivalent weight. **(B)** TPD where each species population is rescaled by its equivalent aboveground biomass in this plot from 1994 to 2020. **(C, D)** Functional richness (FR) of 50% and 99% probability threshold changes over time. The fonts indicate overlap-based functional dissimilarity (β_O_) in different consecutive census periods. The relevant indicators defined as: N_L_, leaf nitrogen concentration; P_L_, leaf phosphorous concentration; N:P_L_, leaf N:P ratio; SLA, specific leaf area; A_sat_, photosynthetic capacity at maximum CO_2_ assimilation rates; *g_s_*, stomatal conductance; *Ks*, sapwood-specific hydraulic conductivity; A_L_/A_S_, leaf area to sapwood area ratio; TLP, water potential turgor loss point; ψ_PD_, predawn leaf water potential; WD, wood density; RC, resource conservation; RA, resource acquisition; HE, hydraulic efficiency; HS, hydraulic safety.

Eleven functional traits based on 345 populations of 69 species were summarized in the first two PC axes (its associated eigenvalues > 1), which together captured 47.03% of variation. The first PC axis associated with all traits explained 29.35% of variation, reflecting the resource acquisitive-conservative trade-off axis. Acquisitive species were linked to lower values of WD and N:P_L_, and higher values of other traits, whereas conservative species exhibited the opposite trait values. The second PC axis explained 17.68% of variation, reflecting the hydraulic safety-efficiency trade-off axis. The negative values indicated species with high hydraulic efficiency, which were strongly related to high photosynthetic capacity (A_sat_ and *g_s_*) and weakly related to high *Ks*, WD, and N:P_L_. The positive values were related to high hydraulic safety (A_L_/A_S_, TLP and ψ_PD_) and leaf indicators (N_L_, P_L_ and SLA) (**Figure 3A**; **Supplementary Tables S2, S3**).

When the trait probability threshold of equivalent weight was rescaled by the AGB per species over the study period, it was observed that 50% functional space was related to biomass-dominant species with high hydraulic efficiency, and only a small space was associated with species with high hydraulic safety. The 50–99% trait space exhibited a broad distribution (**Figure 3B**). The 50% and 99% trait space occupation in 1994, 2004 and 2015 was higher than other years. Notably, the initial severe drought resulted in the highest functional dissimilarities (βo) within all probability thresholds (low overlap trait space), followed by the effects of subsequent drought and windstorm (**Figures 3C, D**).

### Demographic biomass and its trait space in response to climate change

**Figure 4:**
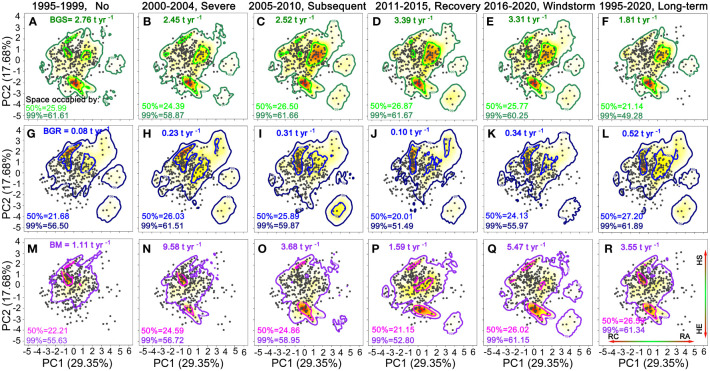
Trait probability densities (TPD) showing the functional trait combinations of each species populations is rescaled by its equivalent biomass growth of survivors (BGS in green colours, **A–F**), biomass growth of recruits (BGR in blue colours, **G–L**) and biomass mortality (BM in purple colours, **M–R**). Functional richness (FR) of 50% and 99% probability threshold in the TPD. RC, resource conservation; RA, resource acquisition; HE, hydraulic efficiency; HS, hydraulic safety.

During the period of high rainfall, BGS (2.76 t ha^–1^ yr^–1^) was significantly higher than BGR (1.11 t ha^–1^ yr^–1^) and BM (0.08 t ha^–1^ yr^–1^) (**Figures 4A, G, M**). Compared to the period of high rainfall, during the initial severe and subsequent drought, BGS significantly decreased by 2.45 and 2.52 t ha^–1^ yr^–1^, while BGR significantly increased by 0.23 and 0.31 t ha^–1^ yr^–1^, and BM significantly increased by 9.58 and 3.68 t ha^–1^ yr^–1^, respectively (**Figures 4D, C, H, I, N, O**). During the recovery period, BGS (3.39 t ha^–1^ yr^–1^) increased significantly, while BGR (1.59 t ha^-1^ yr^-1^) and BM (0.10 t ha^–1^ yr^–1^) decreased significantly (**Figures 4D, J, P**). After the windstorm disturbance, BM, BGS, and BGR reached 5.47, 3.31 and 0.34 t ha^–1^ yr^–1^ respectively (**Figures 4E, K, Q**). From 1995 to 2020, BM, BGS, and BGR were 3.55, 1.81 and 0.52 t ha^–1^ yr^–1^ respectively (**Figures 4F, L, R**).

Compared to the period of high rainfall, BGS covered a narrower trait spectrum space within all probability thresholds during the period of severe drought and windstorm disturbance, but covered a broader trait space during the period of subsequent drought and recovery (**Figures 4A–E**). In contrast, BGR and BM covered a broader trait space within all probability thresholds during the initial severe drought, subsequent drought, and windstorm disturbance, while they covered a narrower trait space during the recovery period (**Figures 4G–K, M–Q**). Long-term BM and BGR covered a broader trait space than BGS (**Figures 4F, L, R**). Yet, BGS and BM were located alongside the hydraulic efficient-safety trade-off axis but only at the high acquisitive side. BGR was located along resource acquisitive-conservative trade-off axis but only on the side associated with high hydraulic safety (**Figure 4**).

**Figure 5:**
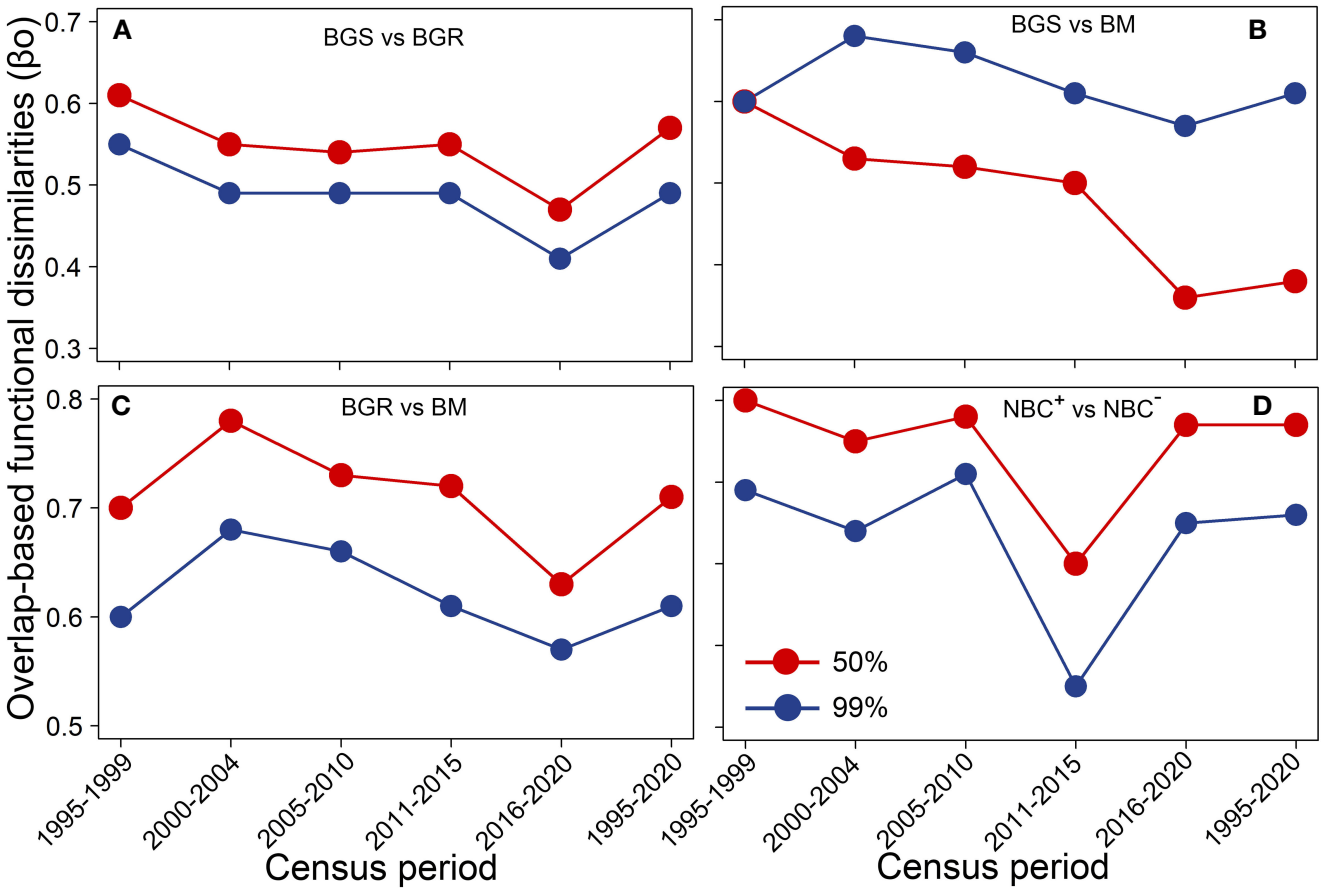
Overlap-based functional dissimilarities (βo) among biomass dimensions **(A–D)** within 50% and 99% probability thresholds during different census periods. BGS, biomass growth of survivors; BGR, biomass growth of recruits; BM, biomass mortality; NBC^+^, positive net biomass changes; NBC^–^, negative net biomass changes.

In 50% threshold trait spectrum space, compared to the period of high rainfall, the initial severe drought, subsequent drought and windstorm events resulted in the trait space occupation of BGS to transform from high hydraulic-efficiency species to higher hydraulic-safety species, whereas that of BM shifted from high hydraulic-safety species towards high hydraulic-efficiency species. The trait space of BGR shifted from conservative to acquisitive species during the drought and windstorm events (**Figure 4**). The βo values among three biomass demographic dimensions declined significantly over time (**Figures 5A–C**). The results of 999 randomizations were consistent with the relative trait space occupancy described above (**Supplementary Table S4**).

### Net positive and negative biomass changes and its trait space in response to climate change

**Figure 6:**
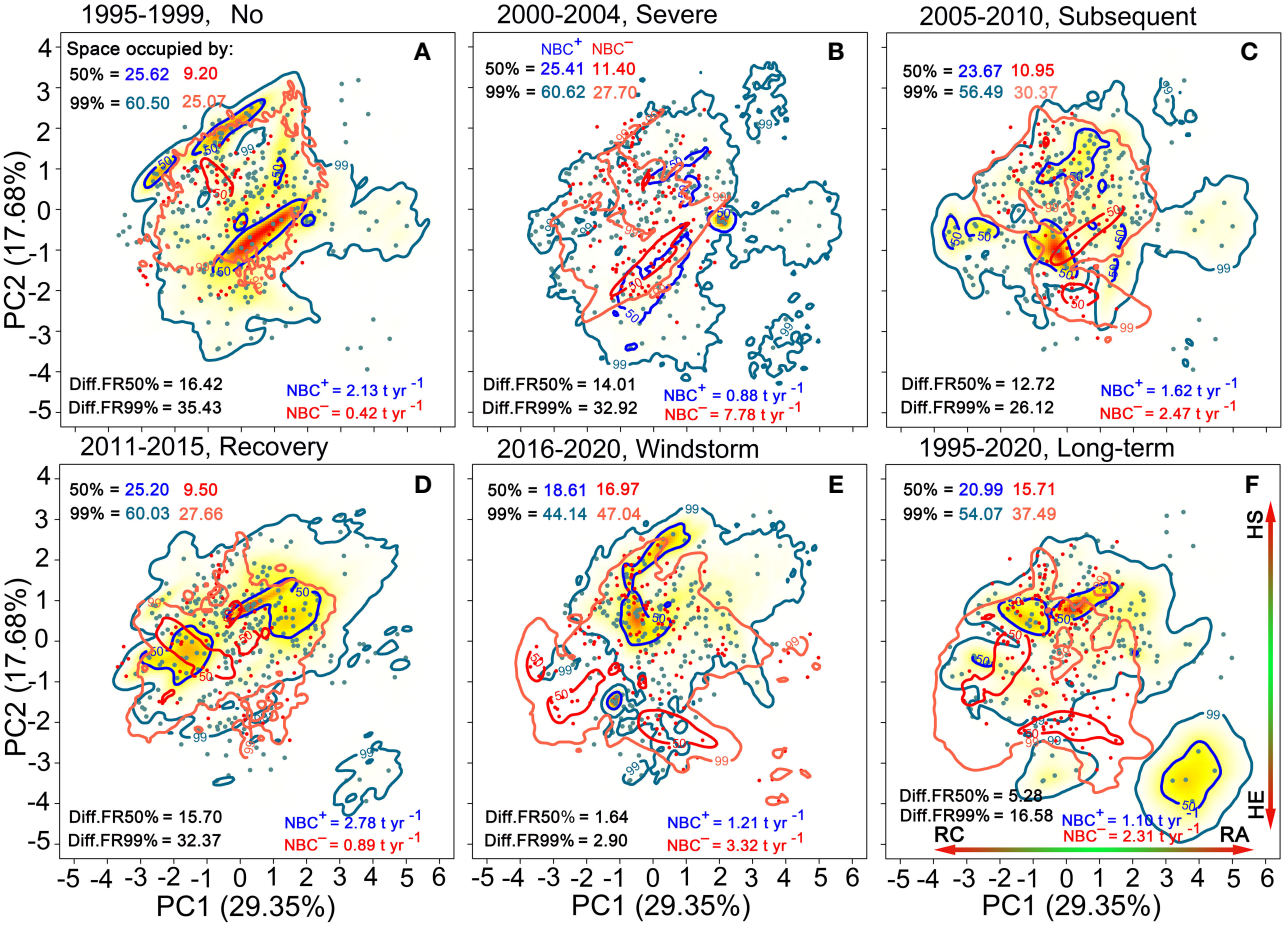
Trait probability densities (TPD) showing the functional trait combinations for species populations is rescaled by positive net biomass changes (NBC^+^ in blue colors) and by negative net biomass changes (NBC^−^ in red colors) **(A–F)**. Functional richness (FR) of 50% and 99% probability threshold in the TPD. Diff.FR refers to differences in FR between NBC^+^ and NBC^−^. RC, resource conservation; RA, resource acquisition; HE, hydraulic efficiency; HS, hydraulic safety.

Compared to the period of high rainfall (NBC^+^ = 2.13 t ha^–1^ yr^–1^) and recovery (NBC^+^ = 2.78 t ha^–1^ yr^–1^), NBC^+^ significantly decreased during the initial severe drought (0.88 t ha^–1^ yr^–1^), subsequent drought (1.62 t ha^–1^ yr^–1^) and windstorm (1.10 t ha^–1^ yr^–1^). However, compared to the period of high rainfall (NBC^−^ = 0.42 t ha^–1^ yr^–1^) and recovery (NBC^−^ = 0.89 t ha^–1^ yr^–1^), NBC^−^ increased significantly during the initial severe drought (7.78 t ha^–1^ yr^–1^), subsequent drought (2.47 t ha^–1^ yr^–1^) and windstorm disturbance (3.32 t ha^–1^ yr^–1^). Long-term NBC^−^ (2.31 t ha^–1^ yr^–1^) was higher than NBC^+^ (1.10 t ha^–1^ yr^–1^) (**Figure 6**).

Compared to the period of high rainfall and recovery, NBC^+^ covered a narrower proportion of trait spectrum space during the period of initial severe drought, subsequent drought and windstorm disturbance, while NBC^−^covered a broader proportion within all probability thresholds. Long-term NBC^+^ covered a broader proportion of trait space than NBC^−^ (**Figure 6**). In particular, NBC^−^ trait space induced by subsequent drought increased continuously compared to the initial severe drought period. Notably, the windstorm-induced NBC^+^ trait space was narrower, while the windstorm-induced NBC^−^ trait space was broader compared to the two drought periods. During the period of initial severe drought, subsequent drought, and windstorm disturbance, the differences in trait space between NBC^+^ and NBC^−^(Diff.FR = |NBC^+^–NBC^−^|) greatly narrowed for all probability thresholds. The windstorm-induced trait space for NBC^−^covered a broader proportion than those for NBC^+^ at 99% probability thresholds. The trait space occupation for NBC^+^ tended to consist of acquisitive species, while that of NBC^−^tended to consist of conservative species with high hydraulic efficiency at 50% probability thresholds (**Figure 6**). Notably, the βo values between NBC^+^ and NBC^−^decreased significantly over time and reached their lowest point during the recovery period (**Figure 5D**).

## Discussion

The impacts of old forests on the global carbon cycle will not be replaceable if they are lost due to global climate change. Here, we used long-term demographic records spanning 26 years and multiple functional traits of 69 coexisting subtropical forest tree species to assess the responses of community biomass dynamics and plant trait spectrum space to climate change. We observed that the occurrence of drought and windstorm events interacting with air warming and elevated CO_2_ reduces biomass gains and increases biomass losses, resulting in more net negative biomass balances. The shifts in functional trait combinations resulting from various climate stresses show new dominant acquisition species with high hydraulic safety gradually emerged and established dominance in the old-growth community. Although compensatory growth was observed during and after drought and storm events, massive biomass loss reduced carbon storage and residence time, ultimately forming a positive carbon-climate feedback loop. Together, in addition to analyzing and expanding demographic biomass dynamics of an old-growth subtropical forest, applying trait-based approaches examines and predicts directional variation of current and future vegetation composition under global climate change. This study makes a substantial contribution to our understanding of community composition dynamics and the drivers of subtropical old-growth forests.

### Climate change increases net negative biomass change

Growing evidence suggests that severe drought is one of the most consequential climatic extremes, exerting a significant impact on the terrestrial carbon cycle (Ciais et al., 2005; Reichstein et al., 2013; Zscheischler et al., 2014). Severe drought may lead to tree mortality directly through hydraulic failure, carbon starvation, or a combination of both (McDowell et al., 2008; Sevanto et al., 2014; Adams et al., 2017), or indirectly through increased vulnerability to forest pests (McDowell et al., 2008; Anderegg et al., 2012; Anderegg et al., 2015a). Severe drought significantly increases tree mortality and biomass loss through limited water availability across species and biomes (Allen et al., 2010; Peng et al., 2011; Anderegg et al., 2015a; Adams et al., 2017; McDowell et al., 2018; van Mantgem et al., 2009; González-M et al., 2020), and it also impacts overall forest structure, biodiversity, and ecosystem function (Anderegg et al., 2012; Anderegg et al., 2015b; Anderegg et al., 2020). Our results demonstrate that the initial severe drought triggers immediate and chronic biomass loss due to tree mortality (**Figures 2A**, **4N**), constrains the biomass growth of survivors (**Figure 4B**), and ultimately increases net negative biomass changes in an old-growth subtropical forest (**Figure 6B**). Furthermore, forest biomass loss remains elevated during subsequent drought and recovery periods because the initial severe drought resulted in numerous cascading indirect effects (**Figures 4O, P**). This indicates that drought-induced mortality can occur immediately or may be delayed, and that biomass loss attributable to tree mortality remains elevated even after drought episode ends, congruent with studies of drought events in the Amazonian rainforest (Aleixo et al., 2019). Severe drought caused by reduced rainfall and warmer air temperatures increases mortality rates for up to two years after the climatic event, synergistically interacting with pathogens, insects, wind throw, and storm damage (Phillips et al., 2010). In particular, large trees are more sensitive to drought stress than smaller trees (Nepstad et al., 2007; Allen et al., 2010; Phillips et al., 2010; Zhou et al., 2013; Zhou et al., 2014; Bennett et al., 2015). Our results highlight that the relative proportions of biomass-dominant species in the old forest under dry conditions have continuously declined since the first census (**Figure 2B**).

Also, strong winds would cause leaf loss, tree collapse, and tree mortality, especially for trees that are less vigorous or have already suffered from droughts, pathogens, and pests (Aleixo et al., 2019; Yee et al., 2019). Our findings suggest that biomass loss caused by windstorm-induced tree mortality exceeds biomass gain produced by survivors and recruits (**Figures 4E, K, Q**), resulting in greater net negative biomass changes in subsequent years (**Figure 6E**). Climatic events cause tree mortality, cumulative physiological and mechanical damage, and the substantial accumulation of leaf litter and woody debris, thereby increasing nutrients available in the soil and potentially benefiting the biomass gained by survivors and recruits (Lodge et al., 1991; Steudler et al., 1991; Suarez and Kitzberger, 2010; Aleixo et al., 2019; Yee et al., 2019). Drought and windstorm events increase the biomass gained by survivors’ growth and facilitate the establishment of recruiting stems for the subsequent recovery period (**Figures 4C–E, I–K**). Increased water availability, climatic warming, and elevated CO_2_ concentrations are expected to enhance plant biomass gain and carbon sequestration in forest ecosystems (Ciais et al., 2005; Brienen et al., 2015; Luo and Chen, 2015; Chen et al., 2016; Hisano et al., 2019). Possibly, the increased growth of surviving trees resulted from the resources released as a result of increased mortality (Brienen et al., 2015; Luo and Chen, 2015). In fact, our results have demonstrated compensatory growth, that is, an increase in biomass growth from survivors and recruits both during and soon after drought and storm events. These mechanisms adequately explain the biomass losses and gains of this old forest in response to various climate change scenarios. Our findings demonstrate that drought and windstorm events increase biomass loss due to tree mortality, decrease biomass gain and even cause significant disruptions to the net carbon balance. These extreme climatic events substantially alter the forest structure and composition, erode the health quality of forest ecosystems, reverse carbon sinks to carbon sources and create positive carbon-climate feedback. That answers the first question we want to solve.

### Climate change reshapes functional trait spectrum space across species

Plant traits associated with the whole-plant economic spectrum suggest that essential patterns of form and function across species can be captured by the two-dimensional spectra of multiple trait combinations (Lavorel and Garnier, 2002; McGill et al., 2006; Westoby and Wright, 2006; Diaz et al., 2015; Laughlin and Messier, 2015; Joswig et al., 2022). As such, quantifying plant trait spectrum space can better capture the breadth and adaptability of tree form and function (Maynard et al., 2022). This old-growth forest across species demonstrates that multiple trait combinations are fundamentally shaped by two main trade-off axes: resource acquisition-conservation and hydraulic safety-efficiency (**Figure 3A**). The widely distributed trait spectrum space across species reveals that greater plant phenotype diversity exhibits multiple alternative combinations and fitness levels under the same environmental conditions. Notably, acquisitive species inherently possess high leaf photosynthetic capacity, stomatal conductance, resource compensation points, capabilities for water and nutrient uptake, and relative growth rates compared to conservative species (Lambers and Poorter, 1992; Sterck et al., 2011). Dominant trees located in the canopy display high hydraulic efficiency, whereas shade- and drought-tolerant smaller trees exhibit high hydraulic safety (Poorter and Bongers, 2006; Prior and Bowman, 2014; Rüger et al., 2018). Likewise, we note that the plant trait spectrum space associated with high-biomass trees tends to include more acquisitive species with high hydraulic efficiency, whereas that related to low-biomass trees contains more conservative species with high hydraulic safety (**Figure 3B**).

The composition and variation of functional traits are shaped by climate variations, resource availability, and disturbance (Feeley et al., 2011; van der Sande et al., 2016; González-M et al., 2020; Lai et al., 2020; Laughlin et al., 2020). Over a 26-year period, climatic perturbations have a pronounced influence on functional trait space occupation in an old-growth subtropical forest. Both during and soon after extreme drought, the trait spectrum space for mortality biomass and recruitment biomass covers a broader range, whereas that for biomass growth covers a narrower range (**Figures 4B, C, H, I, N, O**). Additionally, a broader range of trait space is associated with negative than with positive net biomass changes (**Figures 6B, C**). Similar results have also been observed in tropical dry forests (González-M et al., 2020). During the initial severe and subsequent droughts, biomass loss caused by mortality is strongly negatively associated with net biomass changes (**Supplementary Figures S1B, C**). Prior studies have identified that drought-induced tree mortality is ubiquitous across multiple tree taxa, with less adapted species generating more net negative biomass balances (Allen et al., 2010; Anderegg et al., 2012; Anderegg et al., 2020; González-M et al., 2020). Plant functional traits related to hydraulic damage and stomatal control are useful predictors for the physiological causes of drought-induced mortality (McDowell et al., 2008; Mencuccini et al., 2015; Skelton et al., 2015; Anderegg et al., 2016; Adams et al., 2017). Additionally, the trait spectrum space for windstorm-induced mortality biomass is significantly broader than that for growth and recruitment (**Figures 4E, K, Q**), and even the space of negative net biomass changes is significantly broader than that of positive net biomass changes (**Figure 6E**). Moreover, tall trees with deep crowns and heavy weight are more susceptible to xylem hydraulic failure under water deficits and warming conditions. These trees are also more prone to being affected by windstorms. In this study, high-biomass species with high hydraulic efficiency caused by both drought and windstorm occupy more trait space for tree mortality (**Figures 4N–Q**). While two consecutive drought events increase biomass loss due to selective tree mortality, windstorms cause mortality biomass to cover a wider trait space and threaten more species. The dominant trait space of mortality biomass undergoes a transition from high hydraulic-safety species to high hydraulic-efficiency species, particularly in the presence of drought and windstorm events. This shift signifies the loss of fast-growing pioneer species within this diverse old-growth forest.Selective mortality of specific tree species drives long-term transitions in the dominant species and alters new plant-community assemblages, which can reset or shift successional trajectories (Kreyling et al., 2011; Anderegg et al., 2012). Specifically, biomass growth of surviving trees has a stronger positive correlation with biomass loss due to tree mortality during the initial severe and subsequent droughts, as well as after the windstorm (**Supplementary Figure S2**). It is important to note that widespread rapid mortality events and subsequent alterations could strongly impact tree species and functional composition of survivors and recruits under current climate change, altering the forest composition trajectory of existing communities. Our findings show that drought and windstorm events release stand resource space by removing overstorey trees with high hydraulic efficiency, which could potentially compensate for biomass growth of species with high hydraulic safety. The dominant trait space of biomass growth of survivors undergoes a transition from species with high hydraulic efficiency towards more species with high hydraulic safety (**Figures 4D, G, M**). Eventually, these transitions result in slow-growing tree species being dominant, thereby causing lower carbon sequestration.

Additionally, the dynamic process of tree establishment and growth is highly dependent on recruitment in species-rich communities. Small new recruits suppressed in the understory have a higher tolerance to shade and drought and a lower risk of hydraulic failure under dry conditions. To escape being shaded by competing or neighboring trees, newly established saplings or adult trees tend to grow more rapidly under increased understory light availability (King et al., 2005; Hérault et al., 2011; Iida et al., 2012; Rüger et al., 2018). Tree mortality caused by drought and windstorm events opens up the canopy, creating opportunities for new establishment and growth of understory components. These newly recruited trees with rapid resource acquisition ultimately have greater biomass gains (**Figures 4B, C, E**). We indeed found that biomass recruitment affected by drought and storm disturbance has exceeded that of the undisturbed, indicating a clear case of over-compensation. In combination, positive net biomass changes, consisting of both survivors and recruits, cover less functional trait space and include more acquisitive species with high hydraulic safety, both during and soon after climatic extremes (**Figures 6B, C, E**). Acquisition species with hydraulic safety often have shorter lifespans and higher turnover rates, resulting in faster carbon-water cycling and less carbon storage at the beginning of an extreme event and later in the year. Besides, the functional dissimilarities of multiple demographic dimensions are correlated with the increased availability of resources, such as increased rainfall, warming, and elevated CO_2_ (**Figure 5E**). Climatic extremes interact with subsequent drought, warming, and elevated CO_2_ to reshape function trait space in the forest ecosystem, which reduces carbon storage and residence time. Together, our study has offered a reasonable explanation for biomass composition and dynamics through trait-based approaches, addressing the second scientific question.

## Conclusions

In a species-rich old-growth subtropical forest, the responses of multiple biomass dimensions and their functional trait combinations to multiple climatic scenarios are closely matched to the overall temporal period. Functional trait combinations are associated with the trade-offs between resource acquisition-conservation and hydraulic safety-efficiency. Climatic extreme events synergize with subsequent drought, warming, and elevated CO_2_ to alter long-term biomass dynamics and reshape new dominant trait space. In particular, acquisitive species with hydraulic safety increase biomass gain, whereas acquisitive species with hydraulic efficiency experience greater biomass loss due to increased mortality. In parallel, changing plant trait spectrum space suggests that ongoing climatic changes leads to a shift from being carbon sinks to carbon sources in the old-growth forest, with significant consequences for forest carbon cycle. Based on trait ecology, this study provides a framework to understand the responses of long-term carbon dynamics and vegetation composition changes to various climate change scenarios and underlying mechanism, filling in our knowledge of the forest degradation trajectories and future trends of subtropical forest communities under global climate change.

## Data availability statement

The original contributions presented in the study are included in the article/**Supplementary Material**, further inquiries can be directed to the corresponding author.

## Ethics statement

The manuscript presents research on animals that do not require ethical approval for their study.

## Author contributions

AW: Conceptualization, Investigation, Formal analysis, Methodology, Visualization, Writing – original draft. XX: Conceptualization, Funding acquisition, Project administration, Supervision, Writing – review & editing. RG: Investigation, Formal analysis, Methodology, Visualization, Writing – review & editing. RL, AL, JL, XT and QZ: Investigation, Visualization, Writing – review & editing. All authors contributed to the article and approved the submitted version.
