# Supplementary material for: Climate change reshapes plant trait spectrum to explain biomass dynamics in an old-growth subtropical forest
Source: Front Plant Sci. 2023 Nov 21;14:1260707. doi: 10.3389/fpls.2023.1260707 (PMC10698747; doi:10.3389/fpls.2023.1260707)

**Supporting Information**

**Climate change reshapes plant trait spectrum to explain biomass dynamics in an old-growth subtropical forest**

Anchi Wu^1, 3^, Xin Xiong^2, 3^*, Roy González-M^5,6^ , Ronghua Li^4^, Andi Li^3^, Juxiu Liu^3^, Xuli Tang^3^, Qianmei Zhang^3^

**^1^** Hubei Key Laboratory of Biologic Resources Protection and Utilization, Hubei Minzu University, Enshi, China

^2^ Lushan Botanical Garden, Chinese Academy of Sciences, Jiujiang China

**^3^** Key Laboratory of Vegetation Restoration and Management of Degraded Ecosystem, South China Botanical Garden, Chinese Academy of Sciences, Guangzhou China

^4^College of Natural Resources and Environment, South China Agricultural University, Guangzhou, China

^5^ Programa Ciencias Básicas de la Biodiversidad, Instituto de Investigación de Recursos Biol ógicos Alexander von Humboldt, Cr. 1 # 16-20, Bogotá, Colombia

^6^ Department of Biology, Faculty of Natural Sciences, Universidad del Rosario, Cr. 24 # 63C-69, Bogotá, Colombia

*Correspondence: Xin Xiong xiongx@scbg.ac.cn

**Appendices**

**TABLE S1** Aboveground biomass of 69 species from 1994 to 2020.

| Species | Aboveground biomass (mg ha^–1^) | | | | | |
| --- | --- | --- | --- | --- | --- | --- |
|  | 1994 | 1999 | 2004 | 2010 | 2015 | 2020 |
| *Tarenna mollissima* | 1.29 | 1.89 | 3.93 | 0.00 | 0.00 | 0.00 |
| *Mallotus paniculatus* | 66.96 | 144.98 | 322.11 | 1001.87 | 1963.07 | 1197.61 |
| *Gironniera subaequalis* | 3765.18 | 4875.48 | 6113.75 | 8952.54 | 11897.26 | 12925.99 |
| *Blastus cochinchinensis* | 140.78 | 176.92 | 147.59 | 209.37 | 297.59 | 395.68 |
| *Calophyllum membranaceum* | 3.30 | 2.95 | 0.07 | 0.07 | 0.09 | 0.30 |
| *Meliosma rigida* | 595.12 | 550.34 | 637.85 | 891.25 | 979.00 | 620.39 |
| *Lasianthus chinensis* | 7.61 | 11.68 | 9.07 | 3.23 | 3.47 | 7.34 |
| *Zanthoxylum myriacanthum* | 120.79 | 128.64 | 18.64 | 10.85 | 10.98 | 17.61 |
| *Lindera chunii* | 566.63 | 484.81 | 308.24 | 283.72 | 255.82 | 195.97 |
| *Macaranga sampsonii* | 722.93 | 836.99 | 838.28 | 1824.50 | 2983.70 | 4238.77 |
| *Machilus breviflora* | 103.73 | 119.31 | 127.02 | 136.52 | 138.18 | 2.96 |
| *Pterospermum heterophyllum* | 0.00 | 0.41 | 0.82 | 3.64 | 6.47 | 12.62 |
| *Canarium album* | 2940.29 | 3393.94 | 3490.88 | 3707.24 | 4249.87 | 4483.09 |
| *Memecylon ligustrifolium* | 94.09 | 110.52 | 142.40 | 186.33 | 182.69 | 181.64 |
| *Tsoongiodendron odorum* | 7131.76 | 7399.30 | 7438.02 | 7574.53 | 7772.25 | 7852.76 |
| *Ormosia glaberrima* | 195.74 | 233.44 | 266.72 | 295.16 | 353.95 | 342.84 |
| *Symplocos lancifolia* | 131.59 | 97.34 | 24.25 | 29.44 | 31.38 | 0.00 |
| *Craibiodendron scleranthum* | 1096.25 | 1144.61 | 1222.03 | 1506.62 | 1551.41 | 1649.01 |
| *Bridelia insulana* | 0.59 | 0.59 | 1.78 | 9.92 | 27.83 | 47.18 |
| *Mischocarpus pentapetalus* | 329.12 | 266.46 | 91.86 | 166.00 | 222.41 | 251.83 |
| *Syzygium rehderianum* | 1748.47 | 1928.65 | 1605.05 | 1718.38 | 1945.18 | 1256.85 |
| *Cryptocarya chinensis* | 14797.42 | 14301.15 | 5994.03 | 5481.52 | 3764.60 | 2917.35 |
| *Machilus chinensis* | 3894.05 | 4279.19 | 2120.63 | 2387.80 | 2667.65 | 2807.65 |
| *Cryptocarya concinna* | 21120.45 | 19904.45 | 613.60 | 30.92 | 65.10 | 196.13 |
| *Ficus esquiroliana* | 66.90 | 119.68 | 262.55 | 527.66 | 731.52 | 1042.50 |
| *Engelhardtia roxburghiana* | 5413.12 | 6335.95 | 6423.68 | 2669.99 | 3171.49 | 3397.44 |
| *Xanthophyllum hainanense* | 798.59 | 935.28 | 1104.87 | 1207.62 | 1431.26 | 1699.07 |
| *Sterculia lanceolata* | 1.94 | 3.71 | 4.19 | 9.94 | 24.64 | 47.16 |
| *Chrysophyllum lanceolatum* | 22.89 | 28.57 | 36.96 | 38.30 | 44.83 | 48.46 |
| *Psychotria asiatica* | 240.20 | 285.13 | 267.60 | 150.13 | 94.10 | 63.61 |
| *Archidendron lucidum* | 8.77 | 10.23 | 8.84 | 45.43 | 73.77 | 85.45 |
| *Garcinia oblongifolia* | 232.47 | 262.38 | 305.41 | 227.74 | 11.82 | 6.93 |
| *Macaranga rosuliflora* | 304.50 | 321.23 | 178.47 | 201.85 | 224.69 | 221.30 |
| *Ardisia quinquegona* | 127.99 | 104.81 | 59.74 | 77.31 | 90.48 | 95.54 |
| *Croton lachnocarpus* | 0.00 | 0.50 | 1.19 | 1.47 | 1.73 | 3.35 |
| *Glochidion eriocarpum* | 1.18 | 0.71 | 1.12 | 1.00 | 0.70 | 0.87 |
| *Melastoma sanguineum* | 12.25 | 21.84 | 25.98 | 5.98 | 6.91 | 0.96 |
| *Casearia velutina* | 1.33 | 1.42 | 1.52 | 2.13 | 3.22 | 9.08 |
| *Schima superba* | 16351.79 | 18294.64 | 18601.93 | 17280.41 | 15715.87 | 10774.66 |
| *Antidesma japonicum* | 2.12 | 4.31 | 1.95 | 0.69 | 0.30 | 0.00 |
| *Sarcosperma laurinum* | 457.20 | 523.19 | 667.50 | 931.79 | 983.56 | 769.50 |
| *Evodia lepta* | 8.07 | 6.38 | 0.00 | 3.81 | 0.00 | 1.29 |
| *Ilex chapaensis* | 374.99 | 392.94 | 117.13 | 126.63 | 153.77 | 185.62 |
| *Lindera chienii* | 93.53 | 112.88 | 86.34 | 89.72 | 0.41 | 1.01 |
| *Syzygium levinei* | 179.64 | 178.49 | 187.20 | 228.70 | 251.58 | 180.14 |
| *Acronychia pedunculata* | 697.82 | 643.48 | 570.90 | 599.14 | 494.85 | 198.99 |
| *Nephelium chryseum* | 478.36 | 552.03 | 597.82 | 598.50 | 623.36 | 255.03 |
| *Saurauia tristyla* | 3.00 | 5.70 | 11.11 | 24.26 | 39.78 | 57.01 |
| *Homalium cochinchinense* | 2.67 | 2.67 | 0.26 | 0.78 | 1.88 | 4.48 |
| *Ficus nervosa* | 4.16 | 5.12 | 8.92 | 14.28 | 20.80 | 15.89 |
| *Aquilaria sinensis* | 25.88 | 30.14 | 39.33 | 20.84 | 13.81 | 1.34 |
| *Pygeum topengii* | 4030.73 | 4026.75 | 3823.97 | 2911.97 | 2290.66 | 1855.07 |
| *Diospyros eriantha* | 121.92 | 126.70 | 55.12 | 57.75 | 60.32 | 36.37 |
| *Aidia canthioides* | 260.23 | 317.65 | 424.90 | 828.63 | 1471.71 | 2346.80 |
| *Microdesmis caseariifolia* | 23.56 | 31.79 | 52.64 | 91.41 | 113.58 | 147.60 |
| *Artocarpus styracifolius* | 0.82 | 0.94 | 1.77 | 4.32 | 6.75 | 9.51 |
| *Acmena acuminatissima* | 7607.94 | 8178.19 | 7903.71 | 8517.23 | 9500.77 | 7842.87 |
| *Neolitsea cambodiana* | 18.89 | 22.91 | 25.82 | 33.39 | 10.05 | 24.51 |
| *Schefflera heptaphylla* | 649.04 | 588.44 | 740.28 | 922.60 | 1183.62 | 1451.26 |
| *Canthium dicoccum* | 108.95 | 201.98 | 303.26 | 414.51 | 400.84 | 152.62 |
| *Caryota ochlandra* | 162.60 | 371.09 | 673.37 | 1184.11 | 1436.00 | 1252.95 |
| *Ilex cochinchinensis* | 15.32 | 18.48 | 26.31 | 41.57 | 54.50 | 78.57 |
| *Aporusa yunnanensis* | 6869.41 | 7394.95 | 6553.72 | 5130.07 | 5177.74 | 3751.86 |
| *Pterospermum lanceaefolium* | 1710.43 | 2169.49 | 3071.03 | 4016.93 | 5357.46 | 6120.30 |
| *Ehretia longiflora* | 49.73 | 51.94 | 51.59 | 55.92 | 70.52 | 75.12 |
| *Canthium horridum* | 38.62 | 65.43 | 105.34 | 151.33 | 185.74 | 135.85 |
| *Carallia brachiata* | 1.28 | 2.33 | 4.90 | 8.26 | 19.58 | 45.09 |
| *Castanea henryi* | 68084.06 | 70658.70 | 64404.88 | 58362.71 | 60774.93 | 57495.24 |
| *Syzygium championii* | 10.97 | 10.92 | 19.26 | 27.67 | 31.29 | 26.44 |

**TABLE S2** Bivariate correlation (*r*) between 11 functional traits of 69 tree species in old-growth subtropical forest. Functional traits: leaf nitrogen concentration (N_L_), leaf phosphorous concentration (P_L_), leaf N: P ratio (N:P_L_), specific leaf area (SLA), photosynthetic capacity at maximum CO_2_ assimilation rates (A_sat_), stomatal conductance (*g_s_*), sapwood-specific hydraulic conductivity (*Ks*), leaf area to sapwood area ratio (A_L_/A_S_), water potential turgor loss point (TLP), predawn leaf water potential (ψ_PD_) and wood density (WD).

| Traits | N_L_ | P_L_ | N:P_L_ | SLA | WD | *Ks* | A_sat_ | *g_s_* | A_L_/A_S_ | ψ_PD_ | TLP |
| --- | --- | --- | --- | --- | --- | --- | --- | --- | --- | --- | --- |
| N_L_ | 1 |  |  |  |  |  |  |  |  |  |  |
| P_L_ | 0.53*** | 1 |  |  |  |  |  |  |  |  |  |
| N:P_L_ | 0.04 | -0.71*** | 1 |  |  |  |  |  |  |  |  |
| SLA | 0.30*** | 0.15** | 0.03 | 1 |  |  |  |  |  |  |  |
| WD | -0.19*** | -0.51*** | 0.49*** | -0.18** | 1 |  |  |  |  |  |  |
| *Ks* | 0.22*** | 0.31*** | -0.10 | 0.02 | -0.26*** | 1 |  |  |  |  |  |
| A_sat_ | 0.08 | 0.17** | -0.08 | -0.03 | -0.20*** | 0.61*** | 1 |  |  |  |  |
| *g_s_* | 0.03 | 0.10 | -0.03 | 0.07 | -0.250*** | 0.45*** | 0.80*** | 1 |  |  |  |
| A_L_/A_S_ | 0.37*** | 0.38*** | -0.15** | 0.27*** | -0.41*** | 0.29*** | 0.07 | 0.05 | 1 |  |  |
| ψ_PD_ | 0.10 | 0.07 | -0.06 | 0.27*** | -0.14* | 0.07 | 0.03 | 0.07 | 0.09 | 1 |  |
| TLP | 0.13* | 0.25*** | -0.16** | 0.37*** | -0.27** | 0.06 | 0.04 | 0.04 | 0.13* | 0.22** | 1 |

Significant levels: **P* < 0.05, ** *P* < 0.01, *** *P* < 0.001

**TABLE S3** Factor loading, eigenvalues, the percentage of variance explained and factor scores on the first two principal components. Functional traits: leaf nitrogen concentration (N_L_), leaf phosphorous concentration (P_L_), leaf N: P ratio (N:P_L_), specific leaf area (SLA), photosynthetic capacity at maximum CO_2_ assimilation rates (A_sat_), stomatal conductance (*g_s_*), sapwood-specific hydraulic conductivity (*Ks*), leaf area to sapwood area ratio (A_L_/A_S_), water potential turgor loss point (TLP), predawn leaf water potential (ψ_PD_) and wood density (WD).

| Traits | PC 1 | PC 2 |
| --- | --- | --- |
| N_L_ | 0.60 | 0.34 |
| P_L_ | 0.75 | 0.25 |
| N:P_L_ | -0.34 | -0.06 |
| SLA | 0.23 | 0.52 |
| WD | -0.67 | -0.11 |
| *Ks* | 0.72 | -0.34 |
| A_sat_ | 0.61 | -0.70 |
| *g_s_* | 0.56 | -0.65 |
| A_L_/A_S_ | 0.55 | 0.38 |
| ψ_PD_ | 0.18 | 0.27 |
| TLP | 0.43 | 0.42 |
| Eigenvalues | 3.25 | 1.88 |
| % of variance | 29.35 | 17.68 |
| Cumulative | 29.35 | 47.03 |

**TABLE S4** Functional richness and over-based functional dissimilarity of species populations rescaled by biomass growth of survivors (BGS), biomass growth of recruits (BGR) and biomass mortality (BM) (999 randomisations).

| Census  period | TPD threshold | Functional richness | | | Overlap-based functional dissimilarity (β_O_) | | |
| --- | --- | --- | --- | --- | --- | --- | --- |
|  |  | BGS | BGR | BM | BGS vs BGR | BGS vs BM | BGR vs BM |
| 1995–1999 | 50% | 17.31±1.72 | 14.15±1.66 | 13.70±2.02 | 0.63±0.08 | 0.61±0.08 | 0.68±0.11 |
|  | 99% | 46.47±5.50 | 41.38±5.33 | 40.64±6.04 | 0.58±0.08 | 0.55±0.08 | 0.59±0.11 |
| 2000–2004 | 50% | 16.40±1.61 | 17.05±1.81 | 16.51±1.68 | 0.57±0.09 | 0.53±0.09 | 0.76±0.11 |
|  | 99% | 45.13±5.38 | 46.37±5.48 | 42.96±4.89 | 0.52±0.10 | 0.49±0.10 | 0.69±0.11 |
| 2005–2010 | 50% | 17.70±1.74 | 16.89±1.71 | 16.66±1.73 | 0.57±0.07 | 0.52±0.07 | 0.73±0.09 |
|  | 99% | 46.90±5.36 | 45.22±5.32 | 44.55±5.31 | 0.50±0.09 | 0.48±0.09 | 0.68±0.10 |
| 2011–2015 | 50% | 18.03±1.73 | 12.38±1.67 | 14.12±1.71 | 0.58±0.10 | 0.57±0.10 | 0.72±0.10 |
|  | 99% | 46.97±5.35 | 37.65±5.07 | 39.71±5.07 | 0.53±0.12 | 0.48±0.12 | 0.64±0.11 |
| 2016–2020 | 50% | 17.32±1.63 | 15.3±1.92 | 17.34±1.80 | 0.50±0.09 | 0.37±0.09 | 0.63±0.10 |
|  | 99% | 45.92±5.19 | 42.43±5.31 | 46.4±5.39 | 0.45±0.10 | 0.32±0.10 | 0.57±0.11 |
| 1995–2020 | 50% | 13.81±1.52 | 17.79±1.76 | 17.87±1.77 | 0.63±0.09 | 0.43±0.09 | 0.73±0.08 |
|  | 99% | 38.31±4.03 | 46.95±5.39 | 46.72±5.40 | 0.56±0.10 | 0.39±0.10 | 0.66±0.09 |

**FIGURE S1** Bivariate correlation relationships between three biomass demographic dimensions and net biomass change during different census periods. BGS, biomass growth of survivors; BGR, biomass growth of recruits; BM, biomass mortality.


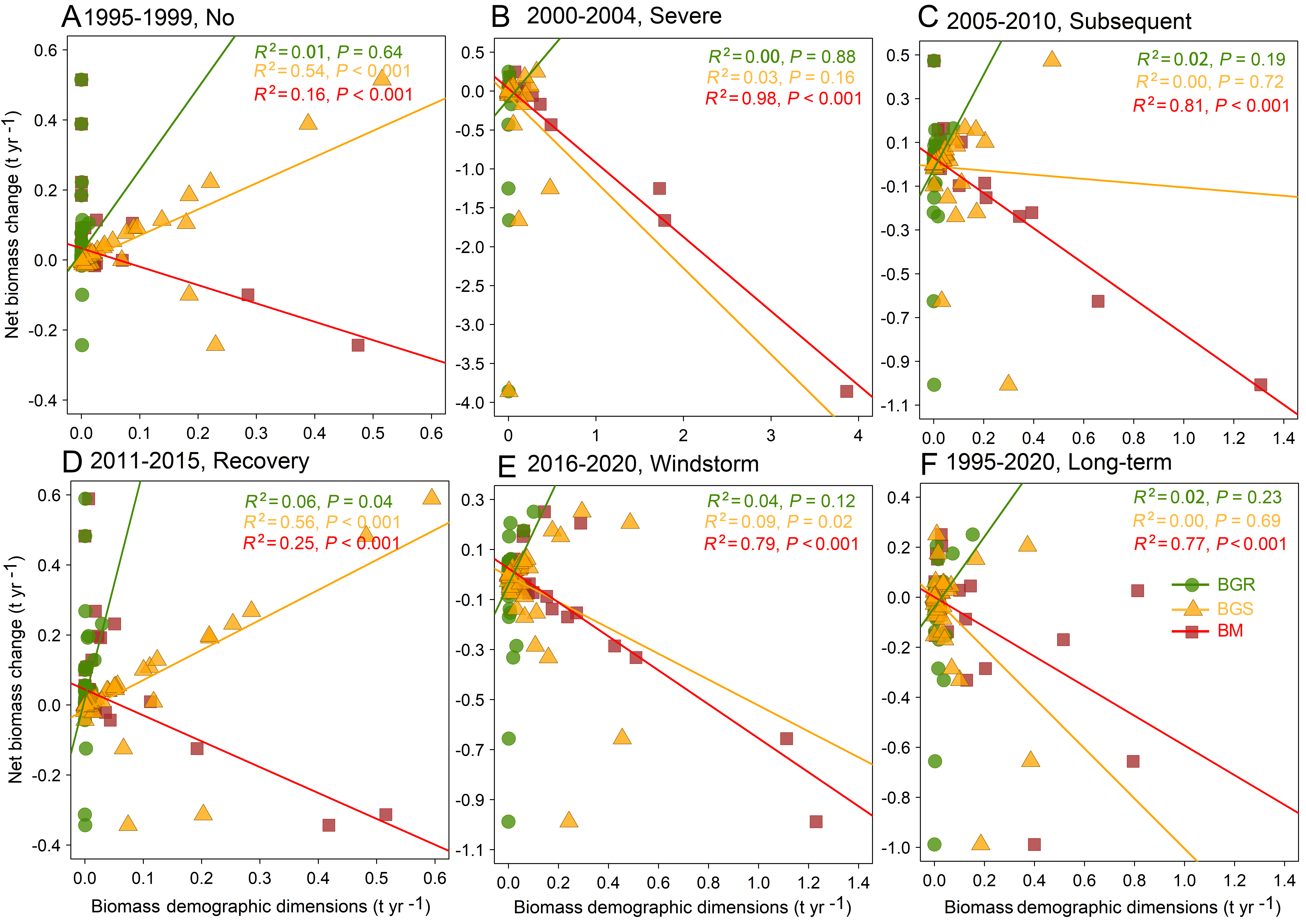


**FIGURE S2** Bivariate correlation relationships between biomass of survivors (BGS), biomass of recruits (BGR) and biomass mortality (BM) during different census periods.


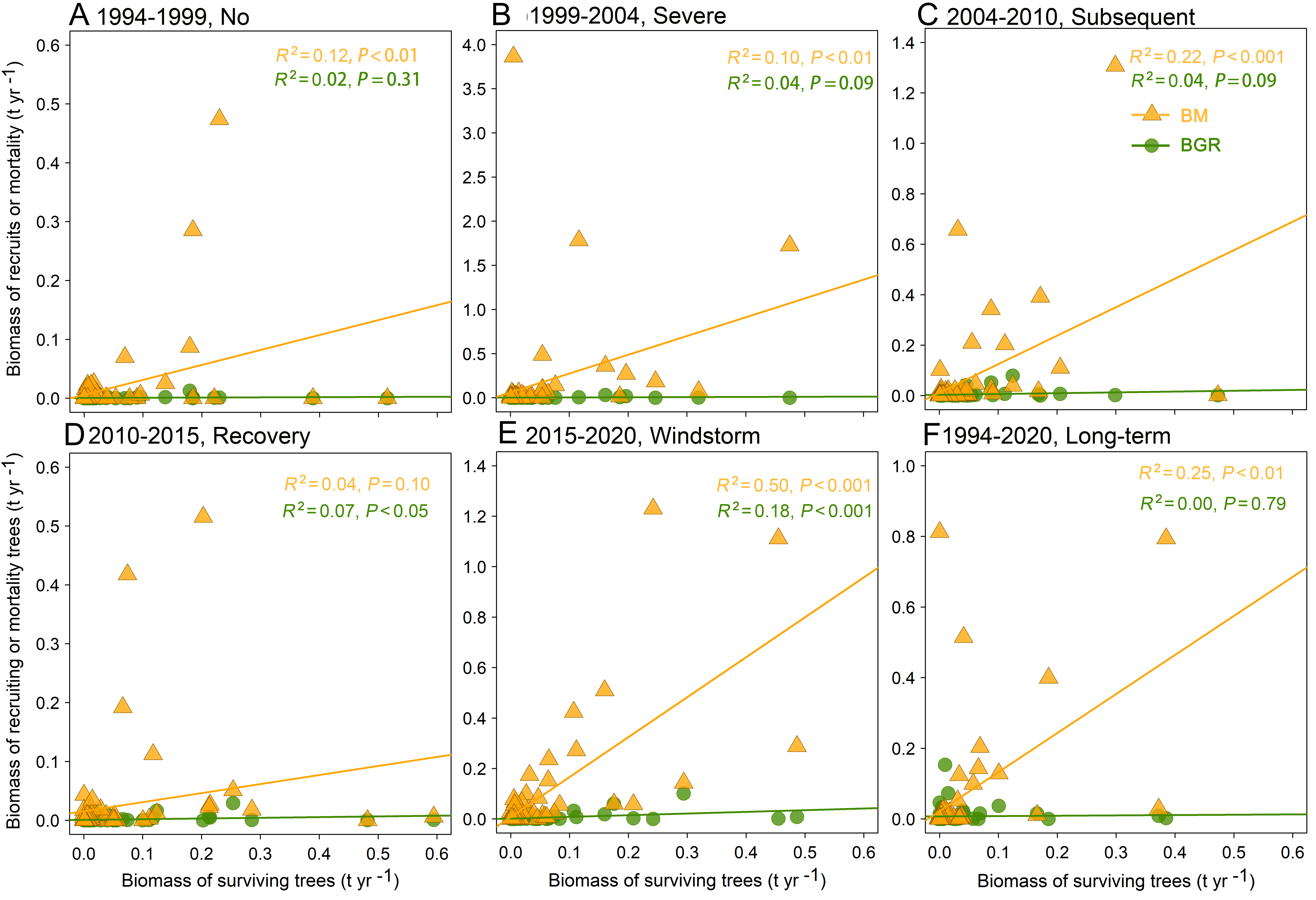

Supplement: Supplementary file 1 [file DataSheet_1.doc]
